# Presentation and survival for urachal cancer: Findings from a nationwide multicenter cohort study in Norway

**DOI:** 10.1177/03915603251358961

**Published:** 2025-08-06

**Authors:** Saima Naz Akhtar, Gigja Gudbrandsdottir, Erling Aarsæther, Birgitte Carlsen, Magne Dimmen, Ingrid Hannestad, Erik Skaaheim Haug, Olav Andreas Hopland, Ann-Karoline Karlsvik, Eirik Kjøbli, Stig Müller, Christian Arvei Moen, Patrick Juliebø-Jones, Christian Beisland

**Affiliations:** 1 Department of Urology, Haukeland University Hospital, Bergen, Norway; 2 Department of Urology, University Hospital in Tromsø, Tromsø, Norway; 3 Department of Pathology, Vestfold Hospital, Tønsberg, Norway; 4 Department of Urology, Bodø Hospital, Bodø, Norway; 5 Department of Urology, Akershus University Hospital, Lørenskog, Norway; 6 Department of Urology, Vestfold Hospital, Tønsberg, Norway; 7 Department of Clinical Medicine, University of Bergen, Bergen, Norway; 8 Institute of Cancer Genomics and Informatics, Oslo University Hospital, Oslo, Norway; 9 Department of Urology, Oslo University Hospital, Oslo, Norway; 10 Department of Urology, St. Olavs University Hospital, Trondheim, Norway

**Keywords:** Urachal cancer, bladder cancer, survival, urachus

## Abstract

**Background and objective:**

This study aims to map the prevalence and treatment of urachal cancer (UrC) in Norway, establish survival rates, identify prognostic factors, and evaluate whether any of the three commonly used staging systems for UrC provide superior prognostic value.

**Methods:**

In this retrospective cohort study, data from the National Cancer Register was collected to identify patients diagnosed with UrC between1997 and2022. Eligible cases (*n* = 43) underwent retrospective review of their individual hospital records. All patients were staged using the Sheldon, Mayo, and Limonnik-revised TNM systems. This was performed locally and then checked by the coordinating center.

**Key findings and limitations:**

The median age at surgery was 59.5 years (IQR 49–73), with 57% of patients being male. The median follow-up time for survivors was 98 months (IQR 81–153). Macroscopic hematuria was the most common presentation (67%, *n* = 28). Recurrence-free survival (RFS) rates at 1, 3, and 5 years were 71%, 57%, and 53%, respectively. Cancer specific survival (CSS) was 95%, 62%, 55%, and overall survival (OS) rates were 93%, 61%, 46% at the same time points. Smaller tumor size was an independent predictor of improved CSS (HR 1.3, CI: 1.01–1.6, *p* = 0.045). Of the three staging systems, only the Mayo system showed statistically significant differences between stages for OS, while none of the systems, including Mayo, showed significant differences for CSS. Study limitations include a small sample size and a prolonged study period of 25 years, which may affect the generalizability of the findings and introduce bias due to changes in clinical practice over time, such as advancements in surgical techniques, and oncological therapies.

**Conclusions and clinical implications:**

Urachal cancer is frequently diagnosed at an advanced stage. Our findings suggest that the Mayo system more effectively distinguishes between localized, locally advanced, and advanced disease compared to the Sheldon and Limonnik-revised TNM systems.

## Background

The urachus is a vestigial remnant of the allantois, which is the main excretory organ of the fetus. Incomplete obliteration during embryogenesis can give rise to a number of congenital anomalies. Furthermore, malignant transformation and urachal cancer (UrC) can occur.^1^ This rare form of bladder cancer is usually diagnosed at an advanced stage, leading to a generally poor prognosis and a 5-year survival rate of approximately 50%.^2^ Given the low incidence of UrC, conducting a clinical study is challenging and no prospective studies have been published to date.^3^ Moreover, there are currently no international guidelines to direct either surgical or oncological treatment. The European Association of Urology (EAU) guidelines on Muscle-invasive and Metastatic Bladder Cancer only briefly mention UrC. However, these recommendations are limited to highlighting that it is key to differentiate urachal and non-urachal subtypes of adenocarcinoma and that an individualized treatment approach is needed in patients with a predominantly non-urothelial histology such as urachal adenocarcinoma.^4^ In recent years, however, rare bladder cancer is being increasingly studied through European consensus efforts and collaborative research initiatives.^3^

For the staging of UrC, several systems have been proposed. Firstly, the Sheldon et al. staging system, which was developed in 1984.^5^ In 1987, the Union for International Cancer Control (UICC) and American Joint Committee on Cancer (AJCC) staging system were combined to form a single TNM staging system, which is still in use today.^6^ In 2006, Ashley et al., proposed the Mayo staging system.^7^ The same year, Pinthus et al. developed the Ontario staging system.^8^ However, the application of that particular system has been very limited. More recently, Limonnik et al., proposed a novel modification to the TNM system for UrC.^9^ However, none of these systems have undergone prospective validation.

Accurate and practical staging systems are essential in clinical practice for treatment planning, patient counseling, and determining eligibility for clinical trials. Research that identifies, which staging system most reliably correlates with survival outcomes can support more consistent and evidence-based patient management. The aim of the study was to assess the clinical outcomes and survival rates for patients treated for UrC in Norway by applying the different staging systems.

## Materials and methods

### Outcomes of interest

Primary outcomes of interest were:

- Prevalence of UrC in Norway
- Clinical treatment of patients diagnosed with UrC in Norway

Secondary outcomes of interest were:

- Survival rates
- Prognostic factors for survival
- Prognostic value among established staging systems (Sheldon, Mayo and Limonnik’s TNM).

### Patient selection and data collection

In this retrospective cohort study, patients with a diagnosis of UrC were eligible for inclusion in the study, and no additional exclusion criteria applied. Patients were identified from the population-based Cancer Registry of Norway (CRN). The CRN provided both the number of all patients registered with a diagnosis of bladder cancer and the number of patients with a registered diagnosis of UrC (*n* = 48) during the period 1997–2022. Since 1953, Norwegian clinicians and pathologists have been legally required to report all new cancer cases to the CRN. All clinical and pathological cancer codes, as well as surgical codes associated with patient-doctor interactions, are automatically transferred from the national public healthcare data systems to the CRN. These codes are cross-checked against clinical and histopathological report forms. If any report forms are missing, reminders are sent to the relevant departments. The CRN is also linked to the Norwegian Population Registry. As of 2024, the registry database contains information on approximately 2 million cancer cases, with coverage close to 100%.^10^

In Norway, advanced bladder cancer surgery is centralized at seven centers. All these centers were contacted to provide data on patients who had undergone surgical treatment during the study period.

Individual hospital records were reviewed at each center, and the relevant data were collected retrospectively. This included patient characteristics, surgery- and histopathology-related variables, as well as data on complications, and treatment after surgery. End of study period was August 1st, 2024.

### Staging

Table 1 provides an overview of the different staging systems utilized, including the Sheldon system, the Mayo system, and Limonnik-revised TNM system (hereafter referred to as TNM). All patients were staged using each of these systems. The original pathology reports were reviewed by the local clinician at each participating center. In some instances, the original report had provided staging according to the Mayo and TNM systems, while the Sheldon staging was performed by the local clinician. Subsequently, two of the authors (GG and CB) independently reviewed the written reports and staging data from all centers to verify accuracy, resolve any discrepancies and ensure that each patient had been staged according to all three systems. Imaging studies were not reviewed again. For the purpose of analysis, the stages were grouped into three categories: localized disease, locally advanced disease, and advanced disease. Table 1 details how the individual stages in each system correspond to these categories. Specifically, TNM stages I–II are classified as localized disease, TNM stage III as locally advanced disease, and TNM stage IV as advanced disease. Tumor size, using a dichotomous 3 cm cut off, based on the EAU guidelines, was included as a covariate in the multivariable Cox regression model to assess its prognostic value. Of note, this value has not been validated for UrC but is drawn from wider data on bladder cancer.

**Table 1.** Staging systems for urachal carcinomas and number of patients in each category (*n* = 42).

Disease status	TNM (Limonnik)—staging system (9)	Mayo staging system (7)	Sheldon staging system (5)
Localized disease	I: Confined to the urachus submucosa (*n* = 3)	I: Confined to the urachus and/or bladder (*n* = 14)	I: Limited to the urachal mucosa (*n* = 0)
	II: Invasion of bladder muscularis propria or microscopic invasion of bladder perivesical tissue (*n* = 13)		II: Invasion confined to the urachus (*n* = 0)
Locally advanced disease	III: Macroscopic invasion of bladder perivesical tissue or invasion of nearby structures, including uterus/vagina/prostate (*n* = 14)	II: Extending beyond the muscular layer of the urachus and/or bladder (*n* = 18)	IIIA: Local extension to the bladder (*n* = 30)
			IIIB: Local extension to the abdominal wall (*n* = 1)
			IIIC: Local extension to the peritoneum (*n* = 2)
			IIID: Local extension to viscera other than bladder (*n* = 2)
Advanced disease	IV: Invasion of the pelvic/abdominal wall/peritoneum or any nodal or distant site involvement (*n* = 12)	III: Infiltrating the regional lymph nodes (*n* = 5)	IVA: Metastases to the regional lymph nodes (*n* = 4)
		IV: Infiltrating non-regional lymph nodes or other distant sites (*n* = 5)	IVB: Metastases to distant sites (*n* = 3)

### Statistical analysis

Descriptive analyses were performed for the patients and tumor characteristics. The Kaplan-Meier method was used for estimation of overall (OS), recurrence free (RFS)- and cancer specific survival (CSS), while differences between groups were assessed using the Log-Rank test. To control for potential confounding factors, multivariable Cox proportional hazards models were used to identify independent predictors of CSS and OS, with relevant clinical, and pathological variables included as covariates. Complete data for the survival estimates with 95% confidence intervals (CIs) is available in the Supplemental Appendix, along with the complete data for the Cox regression analyses.

A *p*-value < 0.05 was considered statistically significant. Statistical calculations were performed using the IBM^®^ SPSS^®^ Statistics software (v29.0) and R (v4.3.3). No *p*-value adjustments were applied.

### Ethics

The Ethics Committee in Western Norway approved this national study (REK-no: 549291), emphasizing that patients should have the ability to withdraw their data if they wished (passive consent). Accordingly, a letter was sent to all living patients with UrC, informing them that their data would be used in the study and giving them the option to opt out. One patient chose to withdraw.

## Results

### Prevalence of UrC in Norway

Between 1997 and 2022, CRN recorded 15,159 cases of bladder cancer, of which 48 were diagnosed with UrC. As a result, UrC represented 0.3% of all bladder cancer cases. For the study, 43 patients with UrC were identified by participating centers and included accordingly. All these patients had undergone surgical treatment. However, one patient later withdrew consent, reducing the final study cohort to 42 patients in total.

### Patients’ characteristics

On August 1st, 2024, the median follow-up for patients still alive was 98 months (interquartile range (IQR) 81–153 months). Median age at surgery was 59.5 years (IQR 49–73), and 57% (*n* = 24) were male. Visible hematuria was the most common presenting symptom (*n* = 28, 67%), in total, 34 patients (81%) were diagnosed based on a symptomatic presentation. Median tumor size was 3.3 cm (IQR 2.0–4.9 cm; Table 2).

**Table 2.** Patients and tumor characteristics.

Parameter	*n* (%)
Median age, years (IQR)	59.5 (49–73)
Sex	
Male	24 (57)
Female	18 (43)
ASA	
1	4 (10)
2	28 (67)
3	7 (17)
4	1 (2)
Missing	2 (4)
Smoking	
Yes	15 (36)
Former	3 (7)
No	23 (55)
Missing data	1 (2)
Median tumor size, cm (IQR)	3.3 (2–4.9)
Symptoms	
Hematuria	28 (67)
Abdominal pain	2 (4)
Dysuria	4 (10)
Non symptomatic	8 (19)
Treatment	
TURBT	2 (4)
Bladder resection	23 (55)
Cystectomy	17 (41)
Complications according to Clavien-Dindo (30 days)	
Total	19 (45)
1	3 (7)
2	9 (21)
3a	1 (2)
3b	5 (12)
Adjuvant treatment	
Chemotherapy	5 (12)
Missing	1 (2)
None	36 (86)
Recurrence	
Local (bladder, lymph node and peritoneal)	10 (24)
Distant (skin, skeletal, and lung)	10 (24)
Treatment of recurrence	
Surgery	5 (12)
Chemotherapy	9 (21)
Radiation therapy	10 (24)
HIPEC	1 (2)
Death (total)	25 (60)
Due to urachal cancer	18 (43)

IQR: interquartile range; HIPEC: hypertherm intraperitoneal chemotherapy; ASA: American Society of Anesthesiologists; TURBT: transurethral resection of bladder tumor.

### Treatment and complications

Most patients underwent partial cystectomy (*n* = 23, 55%), while 17 (41%) underwent cystectomy. Transurethral resection of the bladder tumor (TURBT) alone was performed in two patients (5%).

A total of 45% (*n* = 19) had a postoperative complication within 30 days. The majority of these were Clavian-Dindo (CD) 1–2-(*n* = 12 (28%)). One patient had CD-3a, while five patients had CD-3b, four of whom had intestinal obstruction requiring surgery.

Five patients received adjuvant chemotherapy after surgery. Three of whom had known metastatic disease at the time of surgery and two who underwent partial cystectomy where the final pathology revealed locally advanced disease with tumor growth into the adjacent fat tissue.

### Histopathology and staging systems

According to the Mayo-system, 14, 18, and 10 patients had localized, locally advanced, and advanced disease, respectively. For Sheldon and TNM, the corresponding figures were 0 and 16, 35 and 14, and 7 and 12 (Table 1).

Signet ring pathology was present in 29% (*n* = 12) of cases. Four patients (10%) had positive surgical margins, and seven (17%) had positive lymph nodes on final pathology (Table 3).

**Table 3.** Histopathological features.

Feature	*n* (%)
Positive surgical margins	4 (10)
Positive lymph nodes	7 (17)
Signet ring pathology	12 (29)
Distant metastasis at time of surgery	
Lung	4 (9)
Bone	1 (2)
Other	3 (7)

### Recurrence and survival

**Figure 1.**
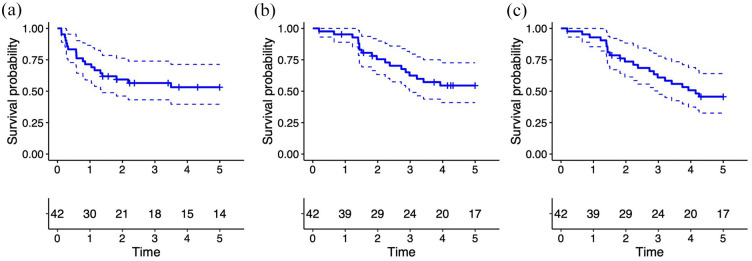
Kaplan–Meier estimates with 95% confidence intervals for (a) 5-year recurrence-free survival, (b) 5-year cancer-specific survival, and (c) 5-year overall survival following treatment for urachal cancer in Norway. The number of patients at risk is shown in the table beneath each respective panel.

Recurrence-free survival rates were 71%, 57%, and 53% at 1, 3, and 5 years, respectively (Figure 1(a)). The median time to recurrence was 9.5 months (IQR 3–17.3). Ten patients (24%) experienced local recurrence, while another ten (24%) developed distant metastases. Subsequent treatment of the recurrences is shown in Table 2.

CSS at 1, 3, and 5 years were 95%, 62%, 55%. OS rates at 1, 3, and 5 years were 93%, 61%, 46%, respectively (Figure 1(b) and (c)). In univariate analysis for CSS, there was no significant difference between the genders (*p* = 0.2). However, OS tended to be better for women (*p* = 0.054).

Furthermore, in univariate analysis, no statistically significant differences in either CSS or OS were observed between patients who underwent partial cystectomy and those who underwent radical cystectomy (*p* = 0.126 and 0.393, respectively). Similarly, no significant differences in CSS or OS were noted between patients presenting with symptoms and those who were asymptomatic (*p* = 0.1 and 0.089, respectively). However, patients with tumors larger than 3 cm exhibited significantly worse CSS and OS (*p* = 0.02 and *p* = 0.048, respectively). In multivariable Cox regression analysis, adjusted for age, gender, ASA score, and symptoms at diagnosis, smaller tumor size was an independent predictor of improved CSS (hazard ratio (HR) 1.3, CI: 1.01–1.6, *p* = 0.045). Similarly, both smaller tumor size (HR 1.4, CI: 1.1–1.7, *p* = 0.005) and female gender (HR 0.32, CI: 0.11–0.90, *p* = 0.031) remained as independent predictors of OS.

**Figure 2.**
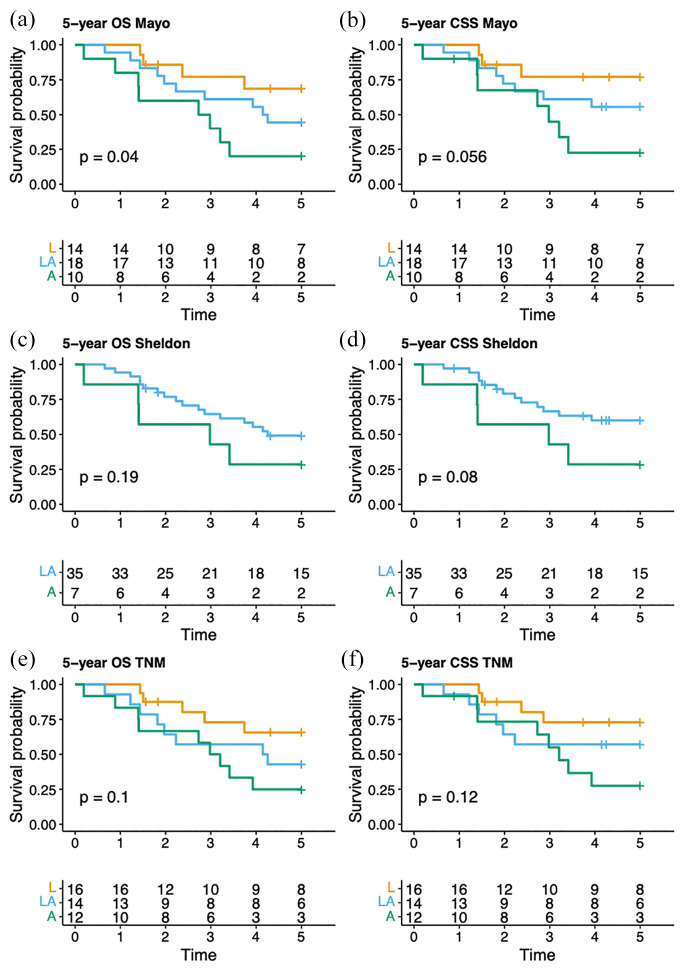
Overall survival and cancer-specific survival estimates up to 5 years after treatment based on the following staging systems: (a and b) Mayo, (c and d) Sheldon, and (e and f) Limonnik’s TNM. The tables below each curve display the number of patients at risk at specific time points. OS: overall survival; CSS: cancer-specific survival; L: localized disease; LA: locally advanced disease; A: advanced disease.

Figure 2 illustrates CSS and OS across the different stages for all three staging systems. The Mayo system demonstrated a statistically significant difference in OS between stages between stages (*p* = 0.04, 2 degrees of freedom), while the difference in CSS was not significant (*p* = 0.056, 2 degrees of freedom; Figure 2(a) and (b)). However, when the stages are tested individually against each other, localized tumors were associated with significantly better CSS and OS compared to advanced disease (*p* = 0.03 and *p* = 0.04 for CSS and OS, respectively). In contrast, no statistically significant differences were observed across stages in the other two staging systems (Figure 2(c)–(f)).

## Discussion

UrC is a rare malignancy, comprising less than 0.2%–0.3% of all bladder cancers.^11^ In this population-based study, we identified 48 cases of UrC between 1997 and 2022, accounting for 0.3% of bladder cancers during the same period. These findings are broadly consistent with a national study from Ireland (1994–2011), which reported 26 cases of urachal cancer, representing 0.3% of all invasive bladder tumors.^12^ Similarly, a study conducted in the Netherlands (1989–2009) identified 152 cases, representing an incidence of 0.2% among all bladder cancers.^13^ The majority of patients in our study were male and older than 50 years. Similarly, this aligns with findings from previous studies conducted in North America,^8,14^ Asia,^15^ and Europe.^12,13^ While these similarities suggest that patterns in UrC may be consistent across these different nations, they should be interpreted with caution as variations in diagnostic thresholds, pathology reporting, and health settings can influence both reported outcomes and patient characteristics.

Recent literature suggests that men with Y chromosome alterations may have an increased risk of developing urological cancers and are associated with poorer clinical outcomes.^16^ Visible hematuria was the main symptom leading to a diagnosis in 67% of cases, a proportion lower than that reported in a previous meta-analysis by Zhang and Wu.^15^ This discrepancy may be attributed to the increasing use of CT imaging performed for other indications, leading to earlier detection in some patients before symptom onset.

Previous studies have not demonstrated a significant survival difference between patients treated with partial cystectomy and those treated with radical cystectomy,^12,13^ and our findings support this observation. However, we found that patients with larger tumors had significantly worse survival outcomes compared to those with smaller tumors (<3 cm). It is likely that treatment selection was influenced by tumor size and other clinical features. This would lead to smaller tumors being more often managed with partial cystectomy, while larger tumors would have required radical cystectomy. This treatment selection bias may account for the lack of observed survival difference between surgical approaches. More invasive procedures, such as radical cystectomy compared to partial cystectomy, are associated with a higher burden of complications. While this must be taken into account, and patients should be informed of these risks as part of the shared decision-making process, the authors believe that tumor size should serve as the primary determinant of the surgical approach. The 5-year OS rate in our study was 46%, comparable to the 45% reported in a nationwide study from the Netherlands by Bruins et al.^13^ Similarly, Dhillon et al.^14^ reported a 46% OS at 62 months in a single-center study at MD Anderson from 1990 to 2010. Ashley et al.^7^ presented a 49% 5-year CSS for 66 patients treated at the Mayo Clinic from 1951 to 2004, which is comparable to the 55% CSS observed in our study. A 2016 meta-analysis by Szarvas et al., which included 24 studies with 1010 UrC patients, reported a 5-year OS of approximately 50%.^2^

UrC lacks a standardized and validated staging system. The first staging system, proposed by Sheldon et al. in 1984, is highly detailed, with eight subgroup categories.^5^ Their study included only five patients, none of whom had stage I or II disease. In 2006, Ashley et al. revised this system and proposed the Mayo staging system, which is simpler with only four categories, and outperformed the Sheldon system in their study.^7^ The TNM classification has also been applied to UrC,^6^ and in 2022, Limonnik et al. proposed a revised TNM system,^9^ comparing it to the Sheldon, Mayo, and traditional TNM systems.

In the present study, the Mayo system demonstrated better discrimination between stage groups, with a statistically significant difference observed for OS (*p* = 0.04) although the difference for CSS did not reach statistical significance (*p* = 0.056). While these results suggest that the Mayo system holds superior prognostic value, the findings need to be interpreted with caution based on the small sample size and borderline p values. From a practical perspective, the Mayo staging system is simpler to use, categorizing the disease into fewer stages. This approach may reduce the risk of misclassification, particularly in a retrospective study. In contrast, the eight stages of the Sheldon system offer more detail, however, the risk of interobserver variability is increased accordingly.

In their study of 152 patients, Bruins et al. successfully differentiated survival using the Sheldon staging system, reporting 15% of patients in stage II, whereas none of our patients were classified in this group,^13^ in a study of 152 patients, successfully differentiated survival using the Sheldon staging system, reporting 15% of patients in stage II, whereas none of our patients were classified in this group. Dhillon et al.,^14^ in their analysis of 46 patients, found that the TNM classification predicted survival better than the Sheldon and Mayo systems.

Most cases of UrC are diagnosed after they invade the bladder mucosa and cause hematuria. Consequently, few patients are identified at an early stage, which renders it difficult for smaller studies, like ours, to distinguish between stages effectively.

There are a number of limitations to acknowledge in this study. The study was retrospective in nature, which may introduce both selection and information bias. The small sample size limits the statistical power of the analyses, particularly in the context of multivariable modeling. The low number of events relative to the number of covariates increases the risk of overfitting in the Cox regression models. The small sample size and low number of events also resulted in wide confidence intervals, which should be noted when interpreting the results. No further analysis on local versus distant recurrences was possible to perform. Pathology assessments were not centralized, potentially leading to interobserver variability. Over the extended study period, numerous changes in clinical practice have occurred, including advancements in imaging techniques, surgical methods, and the availability of oncological treatments, which may have introduced heterogeneity in patient management and outcomes. Only those patients who underwent surgical treatment were included, which could lead to selection bias and render our findings to all patients with UrC less generalizable.

Strengths of this study include its national cohort, population-based design, and long-term follow-up. There is a paucity of studies on UrC, and therefore this study is arguably a valuable addition to this evidence gap. To build on these findings, prospective studies with larger samples are needed to assess whether patients with smaller tumors can be managed less aggressively and to allow for evaluations comparing staging systems. A multicenter study across the Nordic countries has been proposed to further investigate these questions and improve the evidence base for the clinical management of this rare urological malignancy.

## Conclusions

Visible hematuria was the most common presenting symptom in UrC and tumor size appears to be an independent predictor of CSS and OS. While the sample size was small, our findings suggest that the Mayo system may offer better prognostic discrimination for OS than the Sheldon and TNM systems in patients with local, locally advanced and advanced disease.

## Supplemental Material

sj-docx-1-urj-10.1177_03915603251358961 – Supplemental material for Presentation and survival for urachal cancer: Findings from a nationwide multicenter cohort study in Norway Supplemental material, sj-docx-1-urj-10.1177_03915603251358961 for Presentation and survival for urachal cancer: Findings from a nationwide multicenter cohort study in Norway by Saima Naz Akhtar, Gigja Gudbrandsdottir, Erling Aarsæther, Birgitte Carlsen, Magne Dimmen, Ingrid Hannestad, Erik Skaaheim Haug, Olav Andreas Hopland, Ann-Karoline Karlsvik, Eirik Kjøbli, Stig Müller, Christian Arvei Moen, Patrick Juliebø-Jones and Christian Beisland in Urologia Journal
