# Supplementary material for: Presentation and survival for urachal cancer: Findings from a nationwide multicenter cohort study in Norway
Source: Urologia. 2025 Aug 6;92(4):595–602. doi: 10.1177/03915603251358961 (PMC12572362; doi:10.1177/03915603251358961)

Supplementary appendix

- Presentation and survival for urachal cancer

– Findings from a nationwide multicentre cohort study in Norway.

# Supplementary table 1: Kaplan-Meier estimates with 95% Confidence Intervals at 1-, 3-, and 5-years after treatment of Urachal carcinoma

| **Characteristic** | **Survival**  **(1 year)** | **Survival (3 years)** | **Survival (5 years)** |
| --- | --- | --- | --- |
| RFS | 71% (59%, 86%) | 57% (43%, 74%) | 53% (40%, 71%) |
| CSS | 95% (89%, 100%) | 62% (49%, 80%) | 55% (41%, 73%) |
| OS | 93% (85%, 100%) | 61% (48%, 78%) | 46% (33%, 64%) |

RFS – Recurrence free survival, CSS – Cancer specific survival, OS – Overall survival

**Supplementary table 2: Kaplan-Meier estimates for survival with 95% Confidence Intervals at 1-, 3-, and 5-years after treatment according to Mayo`s, Sheldon`s, and Limonnik`s staging systems for urachal carcinomas.**


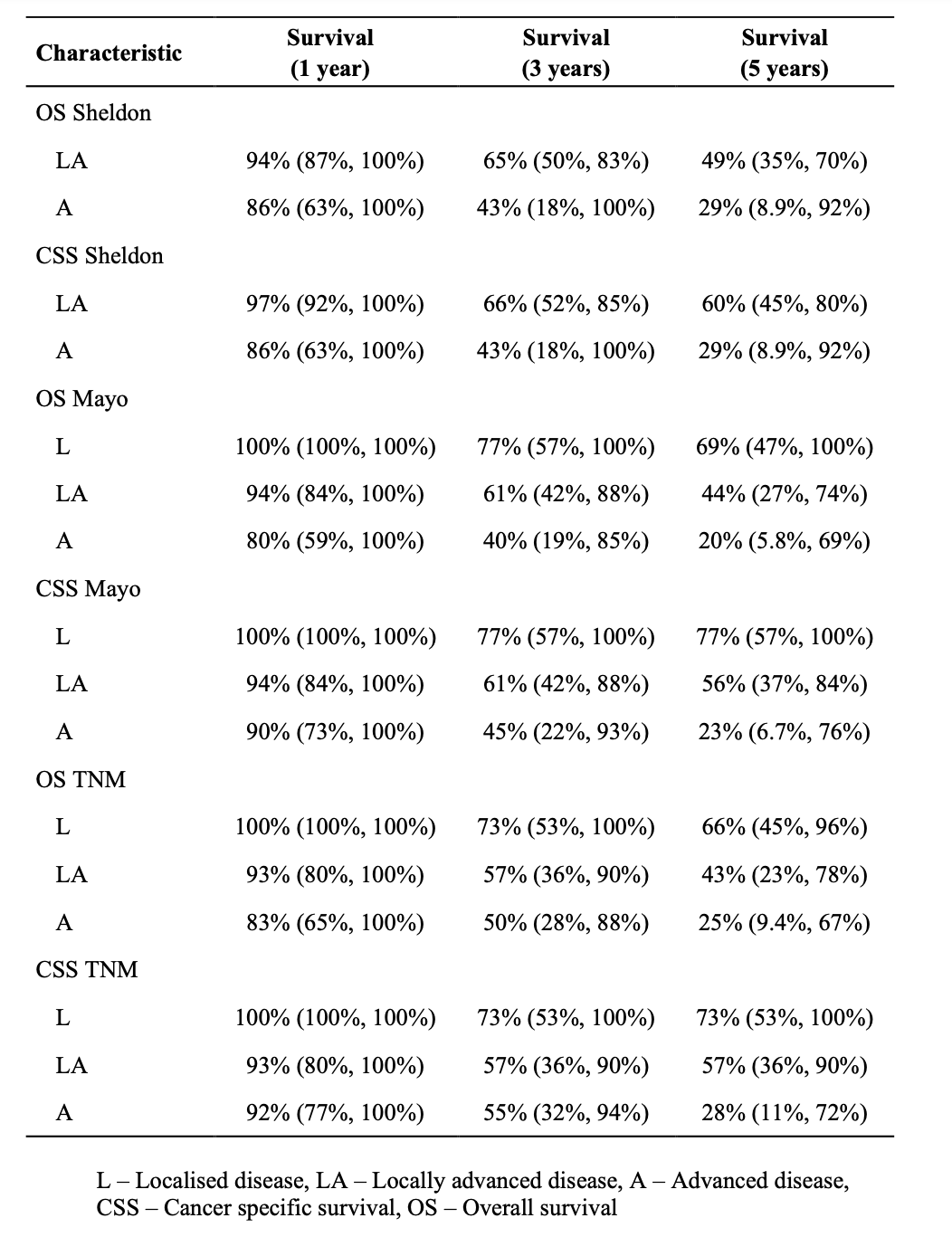


**Supplementary table 3: Cox proportional hazards model for independent predictors for cancer specific survival (CSS)**


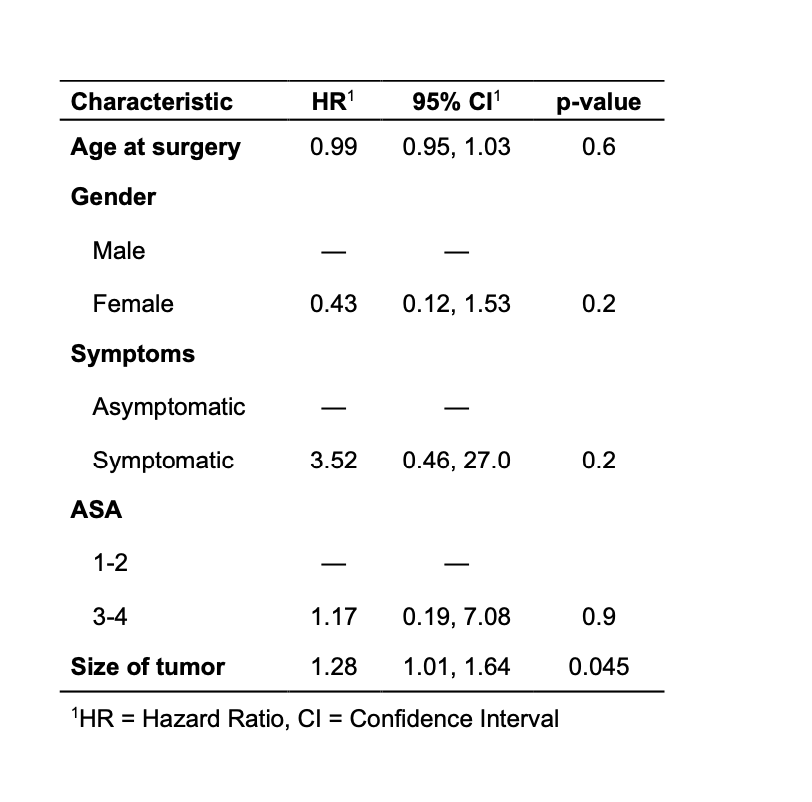


**Supplementary table 4: Cox proportional hazards model for independent predictors for overall survival (OS)**


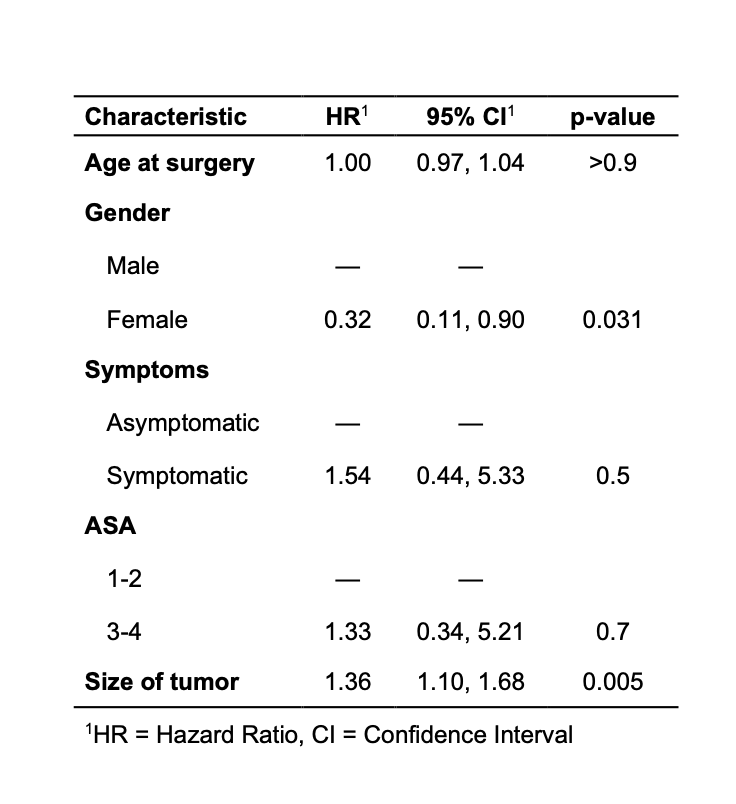

Supplement: sj-docx-1-urj-10.1177_03915603251358961 – Supplemental material for Presentation and survival for urachal cancer: Findings from a nationwide multicenter cohort study in Norway [file sj-docx-1-urj-10.1177_03915603251358961.docx]
